# Stage- and Rearing-Dependent Metabolomics Profiling of *Ophiocordyceps sinensis* and Its Pipeline Products

**DOI:** 10.3390/insects12080666

**Published:** 2021-07-22

**Authors:** Rui Tang, Xue-Hong Qiu, Li Cao, Hai-Lin Long, Ri-Chou Han

**Affiliations:** Guangdong Key Laboratory of Animal Conservation and Resource Utilization, Guangdong Public Laboratory of Wild Animal Conservation and Utilization, Institute of Zoology, Guangdong Academy of Sciences, Guangzhou 510260, China; tangr@giz.gd.cn (R.T.); xhqiu@126.com (X.-H.Q.); caol@giabr.gd.cn (L.C.); hlin_long@163.com (H.-L.L.)

**Keywords:** caterpillar fungus, *Ophiocordyceps sinensis*, *Thitarodes*, metabolites, GC-QTOFMS

## Abstract

**Simple Summary:**

The caterpillar fungus *O. sinensis* is a historical ethnopharmacological commodity in China. The recent die-off of its wild population has raised the urgent task of ensuring the natural conservation of both the fungus and its host species *Thitarodes*. During the last decade, artificial cultivation of the caterpillar fungus has been established to supplement its declining natural colony. However, it is crucial to determine the nutritional background of the artificially reared cordyceps complex and its related products, i.e., mycelia and fruiting bodies reared on media. The current study aims to determine the comprehensive metabolic profiles of 17 treatments from 3 groups, including *O. sinensis* fungus, *Thitarodes* insect and cordyceps complex. We found that the metabolomics of *O. sinensis*-related products were mainly determined by the fruiting bodies rather than the culture methods. Our results suggest that artificially cultured fruiting bodies and cordyceps may have indistinguishable metabolite compositions as the natural ones. These results are highly intriguing in many aspects, from understanding the infection mechanism of caterpillar fungus, to commercial applications of caterpillar fungi quality authentication. It provides insights into the development of better alternatives for wild cordyceps and serves the future biological conservation of the fungal/insect species.

**Abstract:**

Cordyceps, a parasitic complex of the fungus *Ophiocordyceps sinensis* (Berk.) (Hypocreales: Ophiocordycipitaceae) and the ghost moth *Thitarodes* (Lepidoptera: Hepialidae), is a historical ethnopharmacological commodity in China. Recently, artificial cultivation of Chinese cordyceps has been established to supplement the dwindling natural resources. However, much is unknown between the natural and cultivated products in terms of nutritional aspect, which may provide essential information for quality evaluation. The current study aims to determine the metabolic profiles of 17 treatments from 3 sample groups including *O. sinensis* fungus, *Thitarodes* insect and cordyceps complex, using Gas Chromatography - Quadrupole Time-of-Flight Mass Spectrometry. A total of 98 metabolites were detected, with 90 of them varying in concentrations among groups. The tested groups could be separated, except that fungal fruiting body was clustered into the same group as Chinese cordyceps. The main distinguishing factors for the groups studied were the 24 metabolites involved in numerous different metabolic pathways. In conclusion, metabolomics of *O. sinensis* and its related products were determined mainly by the fruiting bodies other than culture methods. Our results suggest that artificially cultured fruiting bodies and cordyceps may share indistinguishable metabolic functions as the natural ones.

## 1. Introduction

Traditional cordyceps consists of the fungus *Ophiocordyceps sinensis* (Berk.) (Order: Hypocreales, Family: Ophiocordycipitaceae), which parasitizes on the caterpillar of *Thitarodes* (Lepidoptera, Hepialidae) and forms a complex. It is endemic at an altitude of 3000–5000 m on the Tibetan Plateau [1]. The complex is a highly regarded health remedy that has been recognized as an ancient herbal medicine. The use of caterpillar fungus as an ethnopharmacological treatment has been extensively reported since the 18th century [2,3,4]. In addition, *O. sinensis* has long been used as an adjuvant cancer therapy in China. In preclinical and clinical research, it showed activity in suppressing tumor growth [5]. Modern medical studies have also shown that the fungus possesses beneficial pharmacological effects, including immunomodulation, antioxidant, anti-apoptosis, renal protection, anti-inflammation and antitumor [6,7,8,9,10,11]. 

The insect hosts for the formation of cordyceps are also known as ghost moths [12]. So far, a total of 54 species of Hepialidae belonging to the genus *Thitarodes* have been reported as insect hosts of *O. sinensis* [13]. They represent a primitive lepidopteran group and evolve from the upstream lineage that led to the heteroneuran Ditrysia. The latter includes most higher moths and butterflies, making the ghost moth a particularly interesting group for studies of lepidopteran evolution [14]. Moreover, the restricted distribution at high altitudes has led to exclusive ecological traits in *Thitarodes* and *O. sinensis*. They live in an environmental condition with characteristic features such as hypoxia, low atmospheric pressure, low temperature and high ultraviolet radiation intensity [15]. Moreover, the prolonged life stage of *Thitarodes* caterpillar is a key factor for the successful infection and development of *O. sinensis* fungi [16]. This ecologically unique multitrophic interaction model has attracted much research attention.

The caterpillar fungus, which occupies a special ecological niche, has become the focus of nature conservation attention in recent decades. Wild cordyceps are threatened with extinction mainly due to climate and overharvest factors [17,18]. Currently, conservative interventions are being introduced in the collection process of wild cordyceps [19]. However, the caterpillar fungus industry is predicted to decline significantly in all classes of suitable areas in Tianshan Mountains, Kunlun Mountains and the southwest Qinghai-Tibetan Plateau [20]. On the other hand, the value per kg of wild cordyceps had reached 140,000 USD, and the corresponding market pipeline still exerts great pressure on its biological conservation [19]. Nevertheless, it may be a difficult challenge to conserve the wild species of both *Thitarodes* and *O. sinensis* in the future, unless functional alternatives for the caterpillar fungus are ready to take place in the market.

One practical solution is to explore indoor rearing of the cordyceps. During the last decade, efforts have been made to establish cultivated *O. sinensis* mycelium and appropriately produced insect fungus products. Mycelial products of *O. sinensis* fungus have been produced by fermentation technology [21]. Artificial cultivation protocols of *O. sinensis* fruiting bodies on rice media have been developed [22,23]. Successful cultivation of the final product, including the inoculation of host caterpillars, induction and development of fruiting bodies from the mummified cadaver, has also been achieved [4,16,24].

However, it is essential to systematically determine the nutritional background of artificially reared cordyceps and their pipeline products such as mycelia and fruiting bodies raised on media. Chemical analysis of company- or laboratory-grown cordyceps commodities can provide a basis for promoting alternatives to natural products, which can satisfy markets without causing further damage to conservation. Analytical methods for the detection of chemicals/molecules in the caterpillar fungus are improving, from liquid chromatography (LC), gas chromatography (GC), mass spectrometry (MS), to combined protocols such as LC-MS and GC-MS [25,26]. Other technologies have also been applied, including UV-visible spectrophotometry and nuclear magnetic resonance (NMR) [27]. GC-MS has been considered one of the most practical and versatile research tools in metabolomics studies for unbiased detection of small metabolites in biological samples. Metabolomic differences have been studies among *O. sinensis* strains, products or cross microbial species, e.g., *Paecilomyces tenuipes* and *Cordyceps militaris* [28,29]. Meanwhile, work has been carried out to identify volatiles in different *O. sinensis* products by various extraction methods [30,31].

So far, non-volatile metabolites based on GC-MS studies have mainly focused on targeted compounds such as sterols, fatty acids and polysaccharides [32,33,34]. Comprehensive metabolites profiling and comparison among *O. sinensis*, *Thitarodes* and cordyceps products are still needed. In this study, we conducted comprehensive metabolic profiling of three categories of samples, including *O. sinensis* fungus, *Thitarodes* insect hosts and natural/reared Chinese cordyceps products using GC-QTOFMS. Metabolite data were analyzed using multivariate principal component analysis (PCA), hierarchical cluster analysis (HCA), Partial Least-Squares Discriminant Analysis (PLSDA) and sparse PLSDA (sPLSDA). We examined similarities and differences in metabolites among the three categories of samples. We screened 25 featured metabolites by checking variable importance in projection (VIP), significant analysis (SAM) and Patternhunter. Finally, we predicted the relevant pathways and targets for the 25 featured metabolites against the *Drosophila* KEGG library.

## 2. Materials and Methods

### 2.1. Chemicals

The chemicals used in the study were chromatographic-grade products. Trichloromethane and methanol were used as extraction solvents (Merck, LiChrosolv, Germany). Trimethylsilyl, trifluoroacetamide, trimethylchlorosilane, methoxyamine hydrochloride and pyridine were used as derivatization solvents (all from J&K Scientific, Shanghai, China). Xylitol, adipic acid and valine (Aladdin, Shanghai, China) were used as internal standards for saccharides, organic acids and amino acids (including other substances). The reagents were stored at 4 °C until testing.

### 2.2. Materials

**Figure 1:**
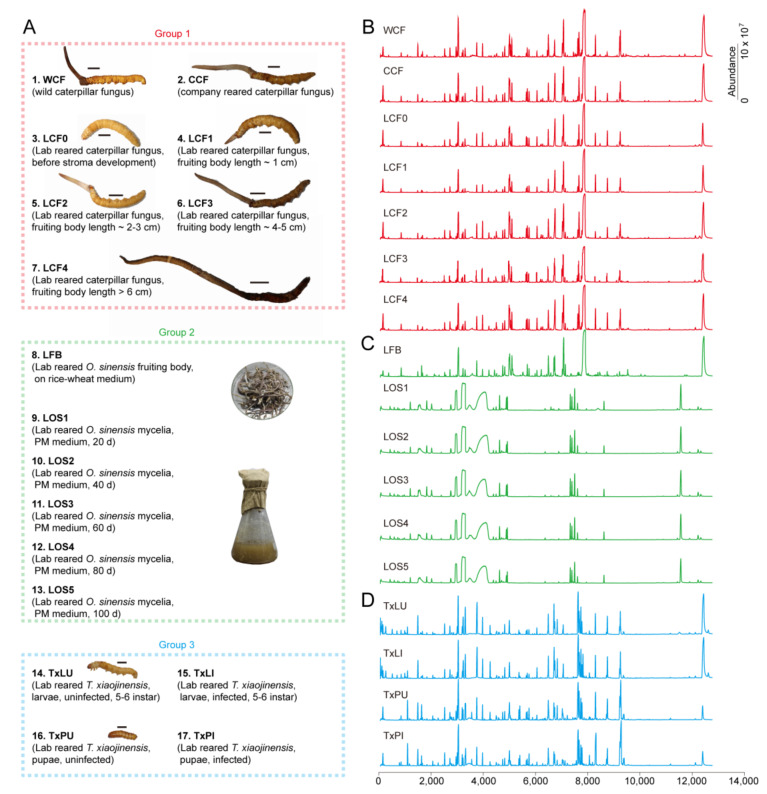
Group setups and example traces of treatments. (**A**) Definition of groups. Group 1 included wild and reared cordyceps. Group 2 included reared fruiting body and mycelia of *O. sinensis*. Group 3 included *T. xiaojinensis* insect host. Detailed descriptions and identifiers for the treatments were provided in Table S1. Each treatment contained n = 50 samples and 3 replicates. (**B**) Traces show treatments of caterpillar fungus (WCF, CCF, LCF0, LCF1, LCF2, LCF3 and LCF4). (**C**) Traces show treatments of reared *O. sinensis* alone (LFB, LOS1, LOS2, LOS3, LOS4 and LOS5). (**D**) Traces show treatments of host insects *T. xiaojinensisi* alone (TxLU, TxLI, TxPU and TxPI).

We used 3 sample groups with 17 treatments for metabolomics analysis (Figure 1A and Table S1). The groups were Chinese cordyceps complex (Group 1), *O. sinensis* fungus alone (Group 2) and *Thitarodes* alone (Group 3).

Group 1 comprised 7 treatments. They were (1) natural cordyceps (Kangding, Sichuan, China) collected and identified according to previous work [23] (WCF); (2) Company cultured cordyceps on *T. xiaojinensis* larvae according to previous works [35] (CCF); (3–7) Lab reared cordyceps of different stages. Culturing was carried out on *T. xiaojinensis* larvae according to previous work [35]. The stages included before stroma development (LCF0), fruiting body length ~ 1 cm (LCF1), fruiting body length 2~3 cm (LCF2), fruiting body length 4~5 cm (LCF3), fruiting body length > 6 cm (LCF4).

Group 2 samples were prepared based on *O. sinensis* KD1223 strains [31]. The corresponding *O. sinensis* strain was preserved at -80 °C in the Institute of Zoology, Guangdong Academy of Sciences, China. Group 2 included 6 treatments. The samples were (8) Laboratory-grown *O. sinensis* fruiting bodies on rice-wheat medium (LFB); (9–13) Laboratory-grown *O. sinensis* mycelia on PM medium during different days. The tested mycelia were grown for 20 d (LOS1), 40 d (LOS2), 60 d (LOS3), 80 d (LOS4) and 100 d (LOS5), respectively.

Group 3 included 4 treatments of *T. xiaojinensis* larvae and pupae. The insect materials were reared at low altitude in Guangzhou under simulated natural conditions according to laboratory protocols [24]. Cytochrome *b* barcodes were used to confirm the insect species [36]. The samples were (14) intact laboratory reared *T. xiaojinensis* larva at 5~6 instar (TxLU); (15) Infected laboratory reared *T. xiaojinensis* larva at 5~6 instar (TxLI); (16) Intact laboratory reared *T. xiaojinensis* pupa (TxPU); (17) Infected laboratory reared *T. xiaojinensis* pupa (TxPU). Infection of the insect was confirmed by checking the hemolymph for blastospores under the microscope prior to the experiments. 

Each sample contained either 50 individuals of caterpillar fungi, mummified cadavers, larvae/pupae or 30 g of fruiting bodies, or 3 flasks of *O. sinensis* with a volume of 150 mL/flask. Each treatment was carried out with 3 biological replicates. Standard protocols and quality control procedures according to previous work were used to prepare each sample prior to chemical analysis [31].

### 2.3. Sample Preparation

All samples were treated with liquid nitrogen before being ground to powder using GT100 mill (Powteq Instrument Co., Beijing, China). Metabolites were extracted with a solvent mixture of methanol, trichloromethane and water in a ratio of 5:2:2 (v/v/v). Here, 100 mg of powder from each treatment was soaked with 3 mL of mixed solvent, vortexed for 1 min and extracted with an ultrasonic bath (RT30, clangsonic Co., Shenzhen, China) for 40 min, then centrifuged under 4000 rpm at room temperature for 5 min. Then the upper layer was collected for further derivatization.

For derivatization, 400 μL of the upper layer was accurately transferred to a GC vial and dried with nitrogen. Then 80 μL of methoxyamine hydrochloride (25 mg/mL, dissolved in pyridine) was added to balance the derivatives of reducing sugars. The mixture was vortexed for 1 min and incubated for 2 h at 40 °C. Then 150 μL of mixed solvent (99% BSTFA plus 1% TMCS, Aladdin, Shanghai, China) was added. The final mixture was vortexed for 1 min and incubated for 1 h at 40 °C. After the samples cooled to room temperature, they were used for GC-QTOFMS analysis.

### 2.4. Chemical Analysis

GC-QTOFMS analysis was performed immediately after the derivatization reactions using an Agilent 7890B-7250 system (Agilent Technologies, Santa Clara, CA, USA) equipped with a HP-5 MS column (30 m × 0.25 mm × 0.25 µm). The sample (0.5 μL) was desorbed in the injection port of the GC at 250 °C. The initial temperature of 50 °C was maintained for 3 min. Then, the oven temperature was increased by 5 °C/min to 280 °C and held at 280 °C for 7 min. The temperature of MS transfer line was set at 280 °C and the temperature of the ion source temperature was set at 200 °C. The ionization potential of MS was 70 eV, the scan range was 50 to 500 m/z and the solvent delay time was 8.0 min.

Data were processed through Agilent Mass Hunter Qualitative Analysis Navigator B.08.00. A NIST 17 library (NIST/EPA/NIH 2017) was used to identify metabolite peaks. The minimum required match factor was 800, and the one-dimensional retention index and exact mass were used to confirm the identified metabolites according to published works [37,38]. A C8 to C25 standard mixture consisting of n-alkanes was used to compare the retention indices between reference chemicals and samples. Internal standard curves were developed using the semi-quantitative method [39]. The relative standard deviation (RSD) reflected the reproducibility of all treatments.

### 2.5. Data Processing

Metabolomic data were analyzed using the MetaboAnalyst 4.0 online pipeline [40]. Retention time and concentration data matrix were imported and generalized log transformation was performed before analysis. Univariate analysis for each metabolite among groups was performed by one-way *ANOVA*. Clustering was performed using Pearson correlation to develop the heat map and dendrogram. Further multivariate analyzes included PCA, PLSDA and sPLSDA which were used to separate the sample groups [41]. Variable importance in projection (VIP), Significance analysis of metabolomics (SAM) and Patternhuntter were used to detect the discriminatory components [42]. Featured metabolites were then used to predict the metabolic pathways involved by Fisher’s exact test against the *Drosophila melanogaster* KEGG library (https://www.genome.jp/kegg/pathway.html) (accessed on 30 September 2020).

## 3. Results

### 3.1. Metabolic Profiles and Overall Assessment

**Figure 2:**
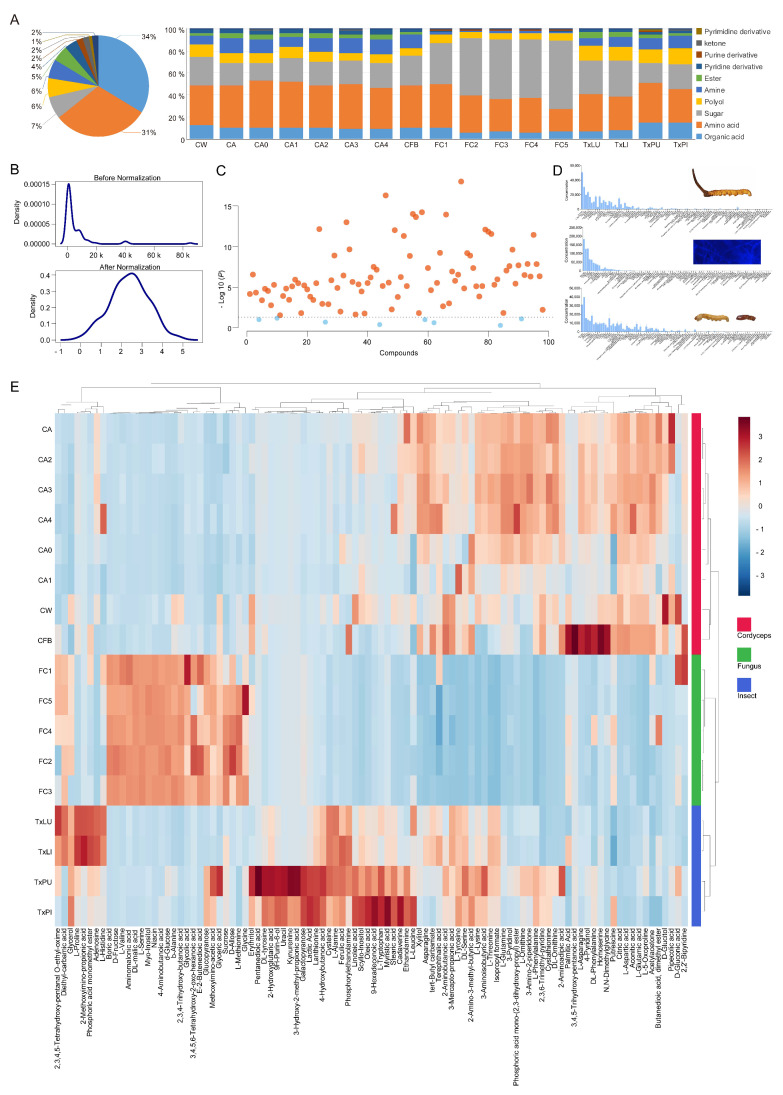
Evaluation of metabolite profiles from the tested treatments. (**A**) The pie chart showed overall proportions of the metabolite classes identified in all products. The bar chart showed the proportions of the same metabolite classes in each treatment from either Chinese cordyceps, *O. sinensis* alone or *Thitarodes* alone. (**B**) Kernel density plots before and after data normalization. Normalization was performed using the generalized log transformation method. (**C**) Comparison of means for the 98 identified metabolites. Most metabolites showed significant differences in concentrations among groups (*ANOVA*, red dots, *p* < 0.05). No significant difference was observed for 8 metabolites including boric acid, erythritol, kynurenine, L-leucine, putrescine, D-gluconic acid, butanedioic acid dimethyl ester and 2,3,4,5-tetrahydroxy-pentanal O-ethyl-oxime (*ANOVA*, blue dots, *p* > 0.05). (**D**) Bar charts showed the concentrations (mg/kg) of 98 metabolites in cordyceps, mycelium and insects. Error bars indicate + SD. (**E**) Heatmap plots showed the distributions of data cells across treatments and metabolites. Distance measurement was by Pearson correlation, and the clustering algorithm was complete.

A total of 98 metabolites were identified from all treatments (Table S2). Samples in each group had similar GC traces, except that the trace from the reared fruiting body was similar to that of the cordyceps group (Figure 1B–D). The identified metabolites included 33 organic acids, 30 amino acids, 7 saccharides, 6 polyols, 6 amines, 5 esters, 4 pyridine derivatives, 2 purine derivatives, 2 ketones, 1 inorganic acid, 1 pyrimidine derivative and 1 thioether (Figure 2A and Table S2). The full list of GC-QTOFMS identified metabolites was summarized, including their corresponding characteristic GC-MS parameters (e.g., retention time, match factor, molecular formula, derivatives of molecular formula, CAS, m/z (M+), m/z (M-CH3) + and mean relative standard deviation (RSD)). All tentatively identified metabolites showed a match factor value above 80. The RSD of all identified metabolites ranged from 5.26% to 16.86%, with an average of 10.93% (Table S2).

The concentrations of metabolites in the different treatment groups were different (Table S3). In general, the dominant metabolites were organic acids, amino acids and saccharides (Figure 2A). Normalization of the data revealed well distributed concentrations of metabolites for further analysis (Figure 2B). Most metabolites showed significant concentration differences among groups, with the exception of boric acid, erythritol, kynurenine, L-leucine, putrescine, D-gluconic acid, butanedioic acid dimethyl ester and 2,3,4,5-tetrahydroxy-pentanal O-ethyl-oxime (Figure 2C). The samples with reared mycelia had the most abundant metabolites with a total amount of more than 6.6 × 10^5^ mg/kg, and they were almost twice as high as the other samples. The highest sugar contents were found in all *O. sinensis* mycelia samples. Sucrose, D-allose, xylitol and 3-pyridinol were the dominant metabolites in all samples with concentrations above 5 × 10^3^ mg/kg (Figure 2D and Table S4). When all metabolites were matched to treatments, standardized abundances of metabolites for the treatments could be separated among groups. In this case, 16 samples showed similar distributions of compounds within groups, with the exception that the reared fruiting body had a similar metabolite distribution to cordyceps (Figure 2E).

### 3.2. Stage, Spatial and Infection Effects

**Figure 3:**
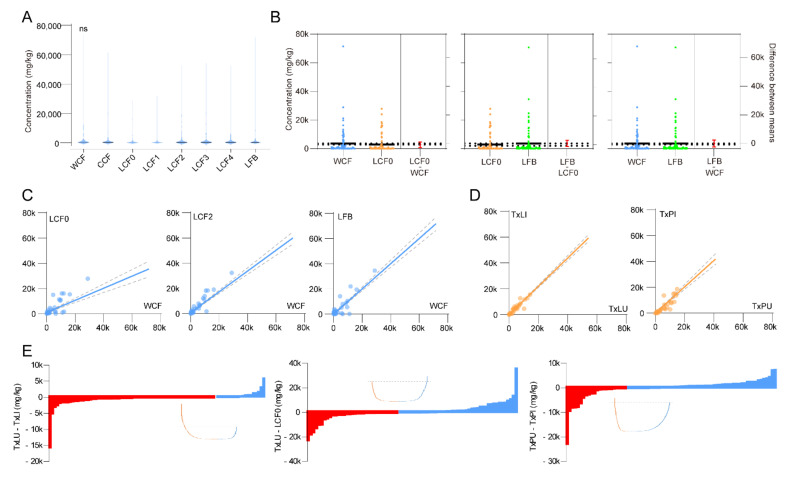
Stage, spatial and infection effectiveness on metabolites. (**A**) Overall comparison of metabolites among WCF, CCF, LCF0~4 and LFB. (*ANOVA*, *F*_7, 776_ = 0.386, *p* = 0.91) (**B**) Estimation plots using *t* tests between selected samples. Color indicates sample category. Error bars indicate S.E.M. No significant difference was observed in 3 tested pairs. (Students’ *t* test, LCF0-WCF: *t*_194_ = 0.79, *p* = 0.43; LCF0-LFB: *t*_194_ = 0.79, *p* = 0.43; LFB-WCF: *t*_194_ = 0.03, *p* = 0.97) (**C**) Linear regression between selected samples for development effectiveness. Dotted lines indicate 95% C. I. Significant regression was found in all pairs. (Linear regression, LCF0-WCF: *F*_1, 96_ = 109.0, *r*^2^ = 0.53, *p* < 0.0001; LCF2-WCF: *F*_1, 96_ = 553.7, *r*^2^ = 0.85, *p* < 0.0001; LCF2-WCF: *F*_1, 96_ = 669.0, LFB: *r*^2^ = 0.87, *p* < 0.0001) (**D**) Linear regression between selected samples for infection effectiveness. Dotted lines indicate 95% C. I. Significant regression was found in all pairs. (Linear regression, TxLI-TxLU: *F*_1, 96_ = 1799, *r*^2^ = 0.95, *p* < 0.0001; TxPI-TxPU: *F*_1, 96_ = 340.4, *r*^2^ = 0.78, *p* < 0.0001) (**E**) Comparison of each metabolite among selected samples for assessment of infection effectiveness. Waterfall plots were developed using minus of selected sample pairs. Red bars indicate up-regulated metabolites, and blue bars indicate down-regulated metabolites.

Total metabolite concentrations showed no significant difference among stages of cordyceps and *O. sinensis* fruiting body but a possible plateau from the LCF2 stage (2–3 cm fruiting body length) was observed (Figure 3A). We specifically compared pairs of wild cordyceps (WCF) and cadavers prior to stroma development (LCF0), LCF0 and fruiting body (LFB), WCF and LFB, without observing a difference (Figure 3B). Trends in metabolite abundances were compared between LCF0 and WCF, LCF2 and WCF, LFB and WCF. During LCF0 stage, a flat but significant correlation was found with WCF (*r*^2^ = 0.53, *p* < 0.0001). While both LCF2 and LFB showed significant positive correlations with WCF samples (LCF2: *r*^2^ = 0.85, *p* < 0.0001; LFB: *r*^2^ = 0.87, *p* < 0.0001) (Figure 3C). This suggests that LCF2 with 2–3 cm fruiting body length is a suitable stage for cordyceps products to be harvested during rearing.

Infection effectiveness was accessed by comparing trends in metabolite abundances between larval treatments (TxLI against TxLU) and pupal treatments (TxPI against TxPU). However, we found that short term infections did not influence the total metabolomics neither in larvae nor in pupae (Larvae: *r*^2^ = 0.95, *p* < 0.0001; Pupae: *r*^2^ = 0.78, *p* < 0.0001) (Figure 3D). We then picked up each metabolite of the above samples to assess infection effectiveness. For larvae, the infection caused up regulations of 65 metabolites, and the top 5 increased metabolites were D-allose, *β*-alanine, phosphorylethanolamine, L-glutamine and oleic acid. Sucrose, L-phenylalanine, putrescine, L-tryptophan and L-leucine were the top 5 down-regulated metabolites by infection. Overall metabolites were increased during short-term infection (Figure 3E). Similar was found in pupae samples, with top 5 up-regulated metabolites of D-glucose, scyllo-inositol, L-leucine, putrescine and oleic acid. The top 5 down-regulated metabolites were *β*-alanine, glycine, DL-serine, phosphorylethanolamine and L-5-oxoproline by infection of pupae (Figure 3E). Long-term infection effectiveness was assessed by comparing TxLU and LCF0. An overall decrease of metabolites was observed, with top 5 up-regulated metabolites of L-glutamic acid, citric acid, L-aspartic acid, L-glutamine and 2-amino-3-methyl-butyric acid. The top 5 down-regulated metabolites in LCF0 were sucrose, glycerol, L-leucine, L-histidine and L-tyrosine, compared to uninfected larvae (Figure 3E).

### 3.3. Clustering and Classification of Treatments

**Figure 4:**
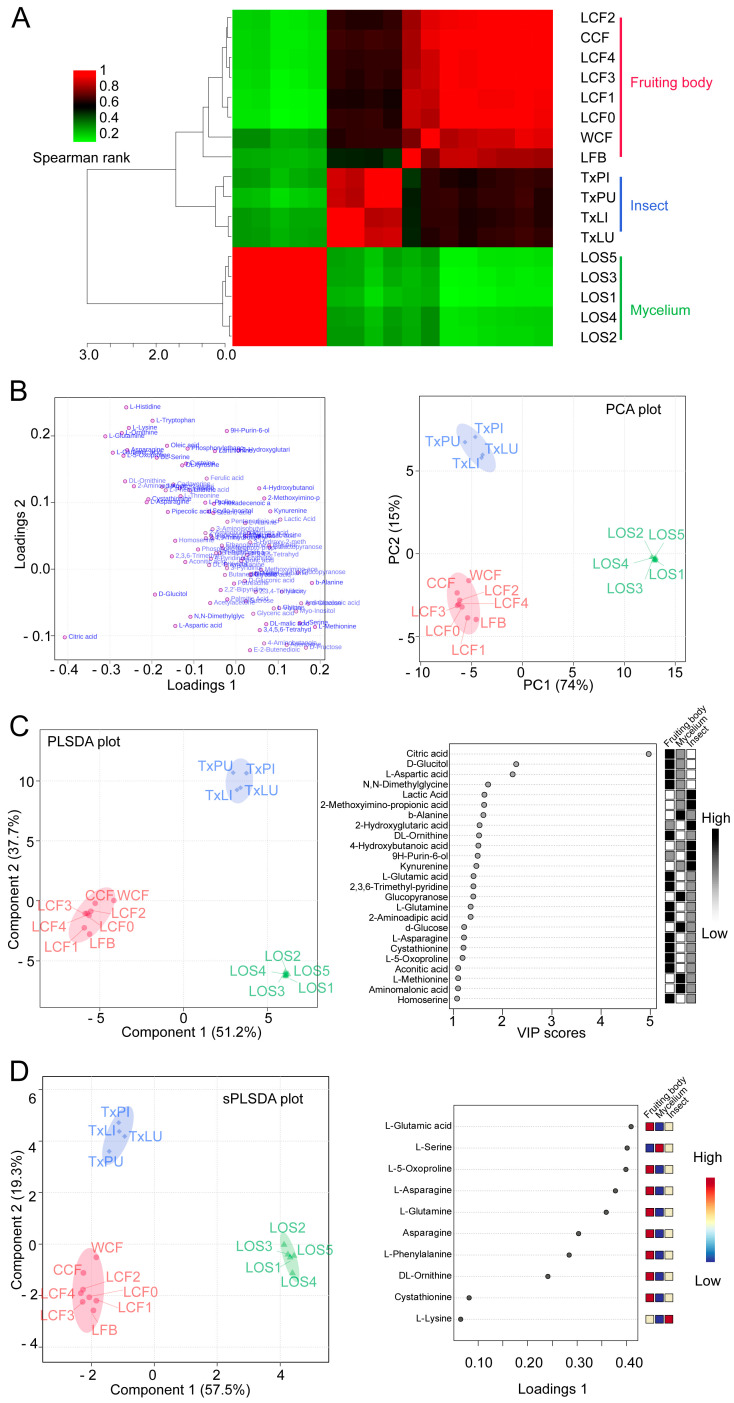
Clustering and group discrimination of samples. (**A**) Correlation matrix of treatments. Dendrogram was obtained using hierarchical clustering from the metabolic profiling in the fruiting body (red), insect (blue) and mycelium (green). (**B**) Loading plot (left) and scores plot (right) from principal component analysis (PCA) between the selected PCs. Circle indicates 95% C.I. (**C**) Partial least squares—discriminant analysis plot (PLSDA, left) and top 25 featured metabolites identified by PLSDA (right). The circle indicates 95% C.I. Boxes on the right indicate the relative concentrations of the corresponding metabolite in each group. (**D**) Sparse PLSDA (sPLSDA) plot (left) and loading plot (right) showing the top 10 variables selected by the sPLSDA model for component 1. The variables are ranked by the absolute values of their loadings. The circle indicates 95% C.I. Boxes on the right indicate the relative concentrations of the corresponding metabolite in each group.

Multivariate analysis was used to test metabolic differences among variables. To obtain a preliminary overview of similarities and differences among groups, pairwise correlations with all 98 metabolites were accomplished. The dendrogram and corrogram showed that the 17 samples were separated into three clusters (Figure 4A). The reared fruiting body (LFB) from merely *O. sinensis* fungus was clustered into cluster cordyceps (Figure 4A). At this point, it makes sense to move LFB treatment into the cordyceps group for further analysis. We then re-organized the groups as (1) fruiting body, (2) insect and (3) mycelium. The new group 1 included cordyceps complex and reared fruiting body because they presented the similar metabolomics profiles (Figure 4A). 

We firstly conducted the PCA test to assess the new clustering. The first 2 principal components (PCs) for plotting presented 89% of the variances. The PCA results did not show overlapping of 95% confidence intervals (C.I.) for all 3 groups. It suggested that the new groups of fruiting body, insect and mycelium were clustered distinctly by their metabolites (Figure 4B). 

We then conducted PLSDA to evaluate the PCA results. The PLSDA scores plot also showed clear separation of the fruiting body of *O. sinensis*, *T. xiaojinensis* insect and mycelium of *O. sinensis* (Figure 4C). PLS (Partial least square) component 1 and component 2 explained 51.2% and 37.7% of the variance, respectively. They added up to 88.9% of the total variance explanation. The VIP value for each metabolite was calculated. Among the 98 ones, 25 metabolites were selected as biomarker metabolites with VIP values > 1 and *p* < 0.05. Among them, citric acid contributed most to the discrimination of the groups, with the highest VIP score at 4.97 to component 1 (Figure 4C).

We further conducted sPLSDA to effectively reduce the number of variables for exploration on easy-to-interpret models. Overview of score plots showed similar separation of treatments as PCA and PLSDA analysis. The score plot was achieved based on 76.8% explanations of variances (component 1 with 57.5% and component 2 with 19.3%) (Figure 4D). Loading plot of top 10 ranked metabolites for group clustering included L-glutamic acid, L-serine, L-5-oxoproline, L-asparagine, L-glutamine, asparagine, L-phenylalanine, DL-ornithine, cystathionine and L-lysine (Figure 4D). 

### 3.4. Selection of Featured Metabolites and Characterizations

**Figure 5:**
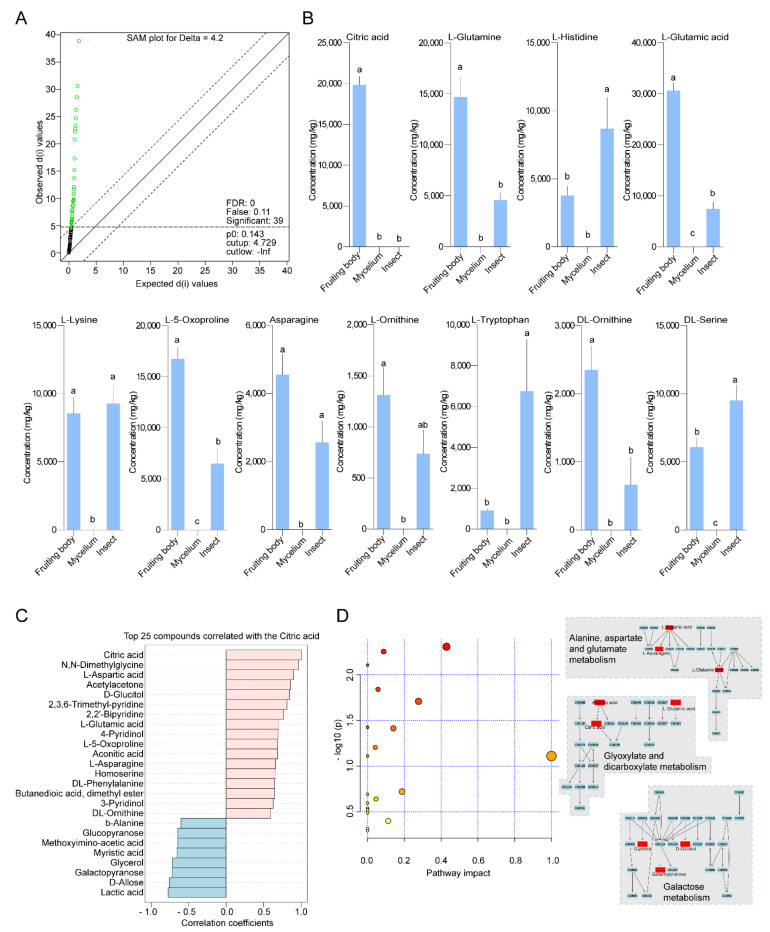
Featured fruiting body metabolites and pathway predictions. (**A**) Significance analysis of metabolomics (SAM) method addressing the false discovery rate (FDR) at Δ = 4.2 to identify featured metabolites. List of featured metabolites was in Table S5. (**B**) Comparison of means in concentrations of the top 11 featured metabolites among groups. Lower-case letters indicate significant differences of concentrations in metabolites observed from groups of fruiting body, mycelium and insect (*GLM* and *Tukey* HSD, *p* < 0.05). (**C**) Top 25 compounds correlated with the citric acid identified by Patternhunter. The selected metabolites represented the most promising metabolites which shared significantly higher abundances in fruiting bodies other than in mycelia or insects. (**D**) Summary of pathway analysis using the metabolites from (**C**). Pathways involving more given metabolites were listed on the right, including alanine/aspartate/glutamate metabolism, glyoxylate/dicarboxylate metabolism and galactose metabolism.

Since all treatments were separated into the corresponding groups in PCA, PLSDA and sPLSDA, replicates for calculation of significance score were assigned to each metabolite by SAM analysis to select featured metabolites from all treatments (Figure 5A). A total of 39 featured metabolites were found with significant variant standard deviations among groups (Table S5). Citric acid was screened as the most variable metabolite and was most abundant in fruiting bodies compared to mycelia and insect hosts (Figure 5B). Furthermore, the top 11 of screened metabolites also showed significant differences among groups in concentrations (Figure 5B). 

We used the Patternhunter algorism to further extract the metabolites that had similar trends with citric acid. The results showed that a total 16 of 98 metabolites presented similar distributions among groups. This suggested that these 16 metabolites were abundant in fruiting bodies (Figure 5C). We observed negative correlation coefficients from 8 of the top 25 metabolites with citric acid. It suggested that these 8 metabolites were concentrated in samples other than fruiting bodies (Figure 5C). 

The 25 featured metabolites were then used in a pathway analysis against the *Drosophila melanogaster* KEGG library. Screened metabolites were predicted to be involved in 20 insect metabolism pathways. Three of them involved most numbers (3 for each) of metabolites (Figure 5D and Table S6). 

## 4. Discussion

We employed the GC-QTOFMS analytical method to investigate metabolites in three representative sample groups of complexes, *O. sinensis* fungus alone and *T. xiaojinensis* insect alone. A total 98 metabolites were identified, and they show distinguishable variations. Multivariate tests showed that all treatments were well-located within the corresponding groups, except that cultured fruiting body was clustered to the cordyceps group. We found it more suitable to divide the sample groups as fruiting body, mycelium and insect, respectively. Featured metabolites which contributed to sample clustering were selected, and citric acid had the highest variation among groups with significantly higher abundance within the fruiting bodies. A total of 16 similar metabolites were selected based on the patterns of citric acid. The results showed that the 25 featured metabolites were involved in 20 different metabolic pathways in insects. 

### 4.1. Situation Dependent Metabolomics of O. sinensis

It is revealed that samples of cadaver prior to stroma development and fruiting body had a similar amount of overall metabolite concentrations. Trends in metabolites from them were similar, too. However, a potential plateau was observed during the development of laboratory cordyceps, which indicates that LCF2 with 2~3 cm fruiting body length was a suitable harvesting target when concerning cost-effectiveness during production. On the other hand, infection of both larvae and pupae had up- and down-regulated certain metabolites. It is possible that these metabolites were involved in post-infection metabolism pathways within the insect-fungus complex [43]. 

Even though each group had several overlapping metabolites at high abundance, treatments can be well separated when tested with total metabolomics profiles. We used HCA, PCA, PLSDA and sPLSDA to discern sample characteristics. We also emphasized on separation of samples according to their origins [44,45,46,47]. The current study revealed an intriguing fact that metabolomics backgrounds of the caterpillar fungus were decided mainly by the fruiting bodies alone. As it showed in the results, we found that reared fruiting bodies of *O. sinensis* had different metabolite profiles from its mycelia samples or its *Thitarodes* hosts. Higher concentrations of metabolites especially saccharides were found in the *O. sinensis* mycelia samples by liquid fermentation rearing. It suggested that *O. sinensis* may absorb various ingredients from the medium and form a consistent proportion of metabolites. This process maintains a steady nutritional background of *O. sinensis* fruiting bodies regardless of how they are produced. In our parallel studies, we found that bacterial and fungal communities varied in wild and laboratory *Thitarodes* lines [48]. Therefore, it is likely that *O. sinensis* may employ innate metabolism pathways to digest the host/medium and produce the fruiting body. It is reported that gene expressions were different during stages of *O. sinensis* fungi, and anamorph mycelia showed large differences from sclerotium or fruiting body stages [49]. Furthermore, glycoside hydrolases were up-regulated in *O. sinensis* fungi after its infection to the *Thitarodes* hosts [49]. This suggested that saccharides were enriched in the mycelia in order to serve as essential nutrients during fruiting body development. Other works also showed that differences were observed in the chemical composition and nutritional properties between natural caterpillar fungus and cultured mycelium of *O. sinensis* [50,51]. The start point of the metabolomics changing in *O. sinensis* fungus during its life stages is still elusive. 

### 4.2. Featured Metabolites and Their Potential Roles as New Quality Control Biomarkers

The metabolomic analytical technique has been used to explore metabolome similarities and differences in *O. sinensis* products or related products. By ^1^H NMR spectroscopy-based approach, metabolic profiles of various products including wild *O. sinensis* from three geographical locations and cultivated mycelia derived from three origins [28], three types of natural *Cordyceps* (*O. sinensis*, *Cordyceps militaris* and *C. nutans*) and *Paecilomyces tenuipe* samples, and 2 types of cultured *C. militaris* samples in aqueous extracts [52], natural *O. sinensis* and commercial *C. militaris* in water-boiled and 50% ethanol-soaked extracts [29] were analyzed. Using LC-MS, the metabolome differences between wild *O. sinensis* and artificial cultured *C. militaris* were conducted [53], and the components of aqueous extract of artificial cultured *C. sinensis* were profiled with focusing on the polar compounds [54]. Previously reported works also concluded that differences in metabolomics existed between mycelia and cordyceps [51]. A few works have focused on development or infection influences. It was revealed that the main life activity metabolism was consistent across *O. sinensis* morphotypes, but each morphotype adopted a different metabolic pattern [55]. It was observed that metabolic changes of *C. militaris* could increase and reach maximal after inoculation with germinated soybean during rearing [56]. In our work, we also observed a potential increase of overall metabolites during stroma development of cordyceps, and this may be used to optimize the cultivation time of relevant products in the future. 

In our previous study, volatile components from three categories of samples, including *O. sinensis* fungi, insect hosts and the cordyceps were analyzed by HS-SPME and GC×GC-QTOFMS, and 119 volatile compounds were identified and the three categories of samples can be separated by PLSDA [31]. The results showed that products from different cultivation methods exhibited different volatile components. Aiming volatiles, we have found that cultured fruiting body was able to be clustered into the group of mycelia but not wild cordyceps. This exhibited a difference between volatile and non-volatile molecules over the development of *O. sinensis* products. Meanwhile, we have also found that gene expressions and microbial compositions were changed during the infection process in *Thitarodes* [57,58]. So far it is agreed that the parasitism process of *O. sinensis* fungus to its host ghost moth has involved complex chemical changes. Additional biomarkers for providing side evidence on quality control are still needed during production [59].

One aim of this study is to select featured metabolites within the caterpillar fungus. By stepwise analytical approaches, a total of 25 enriched biomarkers in fruiting bodies were screened including 9 amino acids, 5 organic acids, 4 pyridine derivatives, 3 saccharides, 2 polyols, 1 ester and 1 ketone. Remarkably, only 4 pyridine derivatives were found amongst tested samples and they were all included in the biomarkers of fruiting bodies of *O. sinensis*. This suggested that these chemicals may be synthesized or concentrated during the fruiting body development. Pyridine derivatives might be key pharmacological elements within this traditional herb. Among them, 3-pyridinol has been classified by MeSH as an antioxidant that can inhibit or retard oxidation reactions in order to counteract the damaging effects of oxidation in animal tissues [60]. Another pyridine derivative 2,2’-bipyridine was classified as a chelating agent. Citric acid as a remarkable indicator of metabolite within the caterpillar fungus was classified as an anticoagulant and calcium chelating agent [60]. Furthermore, N,N-dimethylglycine as the second abundant metabolite in relative concentrations in the caterpillar fungus, was suggested for use as a supplemented diet, immunostimulant and treatment for epilepsy or mitochondrial disease [61,62]. Possible pharmacological ingredients are neither excluded amongst other universal metabolites nor as potential synchronized effects. So far, the exact clinical effects of these selected metabolites would be worth further study to tackle.

Taken together, metabolomics profiles of caterpillar fungus *O. sinensis* and its related products were merely determined by the fruiting bodies rather than culture methods. This indicates that artificially cultured fruiting bodies of *O. sinensis* share indistinguishable non-volatile metabolic traits compared with wild fungus-insect complex. We do not exclude possibilities that large variation may exist in terms of larger molecules, e.g., polysaccharides, and this would worth further study to look into. The current study provided an additional target database that serves quality control of the cordyceps-based drugs.

## Data Availability

All data and visual elements discussed in the current work are provided in the article and online supplementary materials.

## Supplementary Materials

The following are available online https://www.mdpi.com/article/10.3390/insects12080666/s1: Table S1, Treatments and identifiers; Table S2, List of identified metabolites; Table S3, Abundance profiles of identified metabolites; Table S4, Sample-based top ten concentrated metabolites; Table S5, SAM results; Table S6, Pathway predictions of featured metabolites.
