# Supplementary material for: Stage- and Rearing-Dependent Metabolomics Profiling of Ophiocordyceps sinensis and Its Pipeline Products
Source: Insects. 2021 Jul 22;12(8):666. doi: 10.3390/insects12080666 (PMC8396551; doi:10.3390/insects12080666)
Supplement: Supplementary file 1 [file insects-12-00666-s001.zip › insects-1233993-supplementary.pdf]

Supplementary materials for

Stage- and Rearing-Dependent Metabolomics Profiling of *Ophiocordyceps sinensis* and Its Pipeline Products

Contents:

Table S1. Treatments and identifiers.

Table S2. List of identified metabolites.

Table S3. Abundance profiles of identified metabolites.

Table S4. Sample-based top ten concentrated metabolites.

Table S5. SAM results.

Table S6. Pathway predictions of featured metabolites.

Table S1. Treatments and identifiers.

Code	Description	Location	Sample size	Remark
WCF	Wild caterpillar fungus	Kangding, Sichuan	50	
CCF	Company reared caterpillar fungus	Guangzhou, Guangdong	50	Liyuan Co. Ltd
LCF0	Lab reared caterpillar fungus	Lab colony	50	Before stroma development
LCF1	Lab reared caterpillar fungus	Lab colony	50	Fruiting body length ~ 1 cm
LCF2	Lab reared caterpillar fungus	Lab colony	50	Fruiting body length ~ 2 - 3 cm
LCF3	Lab reared caterpillar fungus	Lab colony	50	Fruiting body length ~ 4 - 5 cm
LCF4	Lab reared caterpillar fungus	Lab colony	50	Fruiting body length > 6 cm
LFB	Lab reared *O. sinensis* fruiting body	Lab colony	50	Rice-wheat medium
LOS1	Lab reared *O. sinensis* mycelium	Lab colony	50	PM medium, 20 d*
LOS2	Lab reared *O. sinensis* mycelium	Lab colony	50	PM medium, 40 d
LOS3	Lab reared *O. sinensis* mycelium	Lab colony	50	PM medium, 60 d
LOS4	Lab reared *O. sinensis* mycelium	Lab colony	50	PM medium, 80 d
LOS5	Lab reared *O. sinensis* mycelium	Lab colony	50	PM medium, 100 d
TxLU	Lab reared *T. xiaojinensis* larva	Lab colony	50	Uninfected, 5 - 6 instar
TxLI	Lab reared *T. xiaojinensis* larva	Lab colony	50	Infected, 5 - 6 instar
TxPU	Lab reared *T. xiaojinensis* pupa	Lab colony	50	Uninfected
TxPI	Lab reared *T. xiaojinensis* pupa	Lab colony	50	Infected

* PM medium supplemented with 0.5% *Galleria mellonella* larvae homogenate, 11 ± 2 °C

Table S2. Metabolites identified from different products by GC-QTOF-MS analysis.

Code	Retention time (min)	Family	Compound name	Match factor	Formula	Derivatives of molecular formula	CAS	m/z (M^+)		m/z (M-CH3)$^+$		RSD (%, n=3)
								Theoretical	Experimental	Theoretical	Experimental	
P1	14.17	ketone	Acetylacetone	81	$C_5H1_8O_2$	C_8H_{16}O2Si	13257-81-3	172.0914	172.0916	157.0685		12.19
P2	14.29	Amino acid	N, N-Dimethylglycine	86	$C_4H_9NO_2$	$C_7H_{17}NO_2Si$	1000333-18-4	175.1023		160.0794		11.90
P3	14.37	Organic acid	Methoxyimino-acetic acid	90	$C_3H_5NO_3$	$C_6H_{13}NO_3Si$	55493-91-9	175.0659		160.0430	160.0433	10.41
P4	14.47	Inorganic acid	Boric acid	89	H_3BO_3	$C_9H_{27}BO_3Si_3$	4325-85-3	277.1392		262.1162		12.16
P5	14.48	Pyridine derivative	2,3,6-Trimethyl-pyridine	87	$C_8H_{11}N$	$C_8H_{11}N$	1462-84-6	121.0886	121.0895	106.0656		10.00
P6	15.90	Ester	tert-Butyl carbamate	80	$C_5H_{11}NO_2$	$C_{10}H_{27}NO_2Si_3$	1000366-58-2	277.1344		262.1115	262.1130	12.83
P7	16.38	Organic acid	2-Methoxyimino-propionic acid	84	$C_4H_7NO_3$	$C_7H_{15}NO_3Si$	55493-92-0	189.0816		174.0586	174.0590	12.17
P8	16.53	Organic acid	Diethyl-carbamic acid	87	$C_5H_{11}NO_2$	$C_8H_{19}NO_2Si$	1000322-56-6	189.1180	189.1190	174.0950		8.66
P9	16.74	Organic acid	Lactic Acid	96	$C_3H_6O_3$	$C_9H_{22}O_3Si_2$	17596-96-2	234.1102	234.1171	219.0872		10.47
P10	17.08	Ester	Butanedioic acid, dimethyl ester	80	$C_6H_{10}O_4$	$C_6H_{10}O_4$	106-65-0	146.0574		131.0344	131.0349	11.78
P11	17.18	Organic acid	Glycolic acid	94	$C_2H_4O_3$	$C_8H_{20}O_3Si_2$	33581-77-0	220.0945		205.0716	205.0720	9.86
P12	17.49	Amino acid	L-Valine	87	$C_5H_{11}NO_2$	$C_8H_{19}NO_2Si$	7480-78-6	189.1180		174.0950	174.0947	9.44
P13	18.06	Amino acid	L-Alanine	97	$C_3H_7NO_2$	$C_9H_{23}NO_2Si_2$	27844-07-1	233.1262		218.1032	218.1037	13.44
P14	19.02	Amino acid	Glycine	95	$C_2H_5NO_2$	$C_8H_{21}NO_2Si_2$	7364-42-3	219.1105		204.0876	204.0883	10.64
P15	19.06	Pyridine derivative	3-Pyridinol	82	C_5H_5NO	$C_8H_{13}NOSi$	41571-88-4	167.0761	167.0771	152.0531		6.28
P16	19.23	Organic acid	3-Mercapto-propionic acid	82	$C_3H_6O_2S$	$C_9H_{22}O_2SSi_2$	1000385-85-7	250.0874		235.0644		11.12
P17	19.53	Pyridine derivative	4-Pyridinol	85	C_5H_5NO	$C_8H_{13}NOSi$	1000352-50-3	167.0761	167.0772	152.0531		11.16
P18	19.80	Organic acid	3-Hydroxy-2-methyl-propionic acid	89	$C_4H_8O_3$	$C_{10}H_{24}O_3Si_2$	55530-42-2	248.1258		233.1029	233.1038	9.29
P19	20.19	Amino acid	2-Aminobutanoic acid	94	$C_4H_9NO_2$	$C_{10}H_{25}NO_2Si_2$	55133-91-0	247.1418		232.1189		12.73

Code	Retention time (min)	Family	Compound name	Match factor	Formula	Derivatives of molecular formula	CAS	m/z (M^+)		m/z (M-CH3)$^+$		RSD (%, n=3)
								Theoretical	Experimental	Theoretical	Experimental	
P20	20.39	Ester	Phosphoric acid monomethyl ester	84	CH_5O_4P	$C_7H_{21}O_4PSi_2$	18291-81-1	256.0711	256.0721	241.0481		10.73
P21	21.26	Amino acid	3-Aminoisobutyric acid	97	$C_4H_9NO_2$	$C_{10}H_{25}NO_2Si_2$	58521-52-1	247.1418		232.1189	232.1195	10.23
P22	21.98	Organic acid	4-Hydroxybutanoic acid	86	$C_4H_8O_3$	$C_{10}H_{24}O_3Si_2$	55133-95-4	248.1258		233.1029	233.1038	10.48
P23	22.19	Amino acid	2-Amino-3-methyl-butyric acid	85	$C_5H_{11}NO_2$	$C_{11}H_{27}NO_2Si_2$	1000386-63-4	261.1575		246.1345		14.26
P24	22.68	Amino acid	L-Serine	92	$C_3H_7NO_3$	$C_9H_{23}NO_3Si_2$	70125-39-2	249.1211		234.0981	234.0988	9.93
P25	22.99	Amine	Ethanolamine	96	C_2H_7NO	$C_{11}H_{31}NOSi_3$	5630-81-9	277.1708		262.1478	262.1489	10.59
P26	23.15	Amino acid	L-Leucine	96	$C_6H_{13}NO_2$	$C_{12}H_{29}NO_2Si_2$	7364-46-7	275.1731		260.1502	260.1511	9.12
P27	23.28	Polyol	Glycerol	94	$C_3H_8O_3$	$C_{12}H_{32}O_3Si_3$	6787/10/6	308.1654		293.1424	293.1433	8.30
P28	23.67	Organic acid	Niacin	89	$C_6H_5NO_2$	$C_9H_{13}NO_2Si$	25436-37-7	195.0710		180.0480	180.0487	7.42
P29	23.86	Amino acid	L-Proline	83	$C_5H_9NO_2$	$C_{11}H_{25}NO_2Si_2$	7364-47-8	259.1418		244.1189	244.1195	8.58
P30	24.84	Organic acid	Glyceric acid	92	$C_3H_6O_4$	$C_{12}H_{30}O_4Si_3$	38191-87-6	322.1446		307.1217		8.71
P31	25.01	Pyrimidine derivative	Uracil	95	$C_4H_4N_2O_2$	$C_{10}H_{20}N_2O_2Si_2$	10457-14-4	256.1058	256.1059	241.0828		10.86
P32	25.07	Organic acid	E-2-Butenedioic acid	93	$C_4H_4O_4$	$C_{10}H_{20}O_4Si_2$	17962-03-7	260.0895		245.0665	245.0674	8.77
P33	25.66	Amino acid	DL-Serine	94	$C_3H_7NO_3$	$C_{12}H_{31}NO_3Si_3$	64625-17-8	321.1606		306.1377	306.1396	9.97
P34	26.23	Ester	Isopropyl formate	94	$C_4H_8O_2$	$C_4H_8O_2$	625-55-8	88.0519		73.0289		8.40
P35	26.39	Amino acid	L-Threonine	97	$C_4H_9NO_3$	$C_{13}H_{33}NO_3Si_3$	7537/2/2	335.1763		320.1533	320.1550	9.87
P36	26.62	Organic acid	Pentanedioic acid	89	$C_5H_8O_4$	$C_{11}H_{24}O_4Si_2$	55494-07-0	276.1208		261.0978	261.0983	11.27
P37	26.86	Amino acids	L-Methionine	84	$C_5H_{11}NO_2S$	$C_8H_{19}NO_2SSi$	1000333-29-4	221.0900	221.0902	206.0671		14.05
P38	27.36	Amino acids	β-Alanine	96	$C_3H_7NO_2$	$C_{12}H_{31}NO_2Si_3$	55255-77-1	305.1657		290.1428	290.1442	9.42
P39	27.86	Pyridine derivative	2,2'-Bipyridine	83	$C_{10}H_8N_2$	$C_{10}H_8N_2$	366-18-7	156.0682	156.0692	141.0452		11.00
P40	27.98	Amino acid	Homoserine	87	$C_4H_9NO_3$	$C_{13}H_{33}NO_3Si_3$	1177129-58-6	335.1763		320.1533	320.1549	13.42
P41	28.16	ketone	3-Amino-2-piperidone	81	$C_5H_{10}N_2O$	$C_{11}H_{26}N_2OSi_2$	32565-12-1	258.1578	258.1594	243.1349		13.01

Code	Retention time (min)	Family	Compound name	Match factor	Formula	Derivatives of molecular formula	CAS	m/z (M^+)		m/z $(M-CH3)^+$		RSD (%, n=3)
								Theoretical	Experimental	Theoretical	Experimental	
P42	28.55	Organic acid	Aminomalonic acid	95	$C_3H_5NO_4$	$C_{12}H_{29}NO_4Si_3$	1000079-52-5	335.1399		320.1169	320.1185	12.57
P43	28.99	Organic acid	DL-malic acid	95	$C_4H_6O_5$	$C_{13}H_{30}O_5Si_3$	38166-11-9	350.1396		335.1166	335.1182	8.85
P44	29.60	Polyol	Erythritol	86	$C_4H_{10}O_4$	$C_{16}H_{42}O_4Si_4$	18547-29-0	410.2155		395.1925		8.45
P45	29.80	Amino acid	L-Aspartic acid	89	$C_4H_7NO_4$	$C_{13}H_{31}NO_4Si_3$	55268-53-6	349.1555	349.1578	334.1326		13.52
P46	29.88	Amino acid	L-5-Oxoproline	93	$C_5H_9NO_3$	$C_{11}H_{23}NO_3Si_2$	30274-77-2	273.1211	273.1226	258.0981		9.18
P47	29.99	Amino acid	4-Aminobutanoic acid	93	$C_4H_9NO_2$	$C_{13}H_{33}NO_2Si_3$	39508-23-1	319.1814		304.1584	304.1597	9.52
P48	30.39	Amino acid	DL-Phenylalanine	92	$C_9H_{11}NO_2$	$C_{12}H_{19}NO_2Si$	2899-42-5	237.1180		222.0950	222.0953	9.57
P49	30.66	Amino acid	Cysteine	91	$C_3H_7NO_2S$	$C_{12}H_{31}NO_2SSi_3$	1000153-02-1	337.1378		322.1148		16.86
P50	30.91	Organic acid	2,3,4-Trihydroxy-butanoic acid	92	$C_4H_8O_5$	$C_{16}H_{40}O_5Si_4$	1000352-53-3	424.1947		409.1718	409.1741	11.02
P51	31.07	Organic acid	2-Hydroxyglutaric acid	91	$C_5H_8O_5$	$C_{14}H_{32}O_5Si_3$	55530-62-6	364.1552		349.1323	349.1345	10.08
P52	31.57	Amino acid	L-Asparagine	94	$C_4H_8N_2O_3$	$C_{10}H_{24}N_2O_3Si_2$	1000333-25-8	276.1320	276.1332	261.1090		13.08
P53	31.77	Organic acid	Pipecolic acid	85	$C_6H_{11}NO_2$	$C_{12}H_{27}NO_2Si_2$	55255-44-2	273.1575		258.1345		9.90
P54	32.03	Amino acid	L-Ornithine	80	$C_5H_{12}N_2O_2$	$C_{14}H_{36}N_2O_2Si_3$	24595-70-8	348.2079	348.2106	333.1850		12.74
P55	32.15	Amino acid	L-Glutamic acid	95	$C_5H_9NO_4$	$C_{14}H_{33}NO_4Si_3$	15985-07-6	363.1712	363.1742	348.1482		12.57
P56	32.38	Amino acid	L-Phenylalanine	98	$C_9H_{11}NO_2$	$C_{15}H_{27}NO_2Si_2$	7364-51-4	309.1575		294.1345	294.1359	7.63
P57	33.15	Organic acid	3,4,5-Trihydroxy-pentanoic acid	88	$C_5H_{10}O_5$	$C_{17}H_{42}O_5Si_4$	1000149-61-6	438.2104		423.1874		11.12
P58	33.41	Amine	Asparagine	95	$C_4H_8N_2O_3$	$C_{13}H_{32}N_2O_3Si_3$	55649-62-2	348.1715	348.1740	333.1486		11.25
P59	33.76	Sugar	2,3,4,5-Tetrahydroxy-pentanal O-ethyl-oxime	85	$C_7H_{15}NO_5$	$C_{19}H_{47}NO_5Si_4$	1000394-64-9	481.2526		466.2296		10.90
P60	34.34	Organic acid	2-Aminoadipic acid	87	$C_6H_{11}NO_4$	$C_{15}H_{35}NO_4Si_3$	66434-50-2	377.1868	377.1897	362.1639		11.81
P61	34.73	Polyol	Xylitol	92	$C_5H_{12}O_5$	$C_{20}H_{52}O_5Si_5$	14199-72-5	512.2656		497.2426		5.26
P62	34.90	Amine	Putrescine	96	$C_4H_{12}N_2$	$C_{16}H_{44}N_2Si_4$	39772-63-9	376.2576		361.2347	361.2366	12.92
P63	35.01	Organic acid	Aconitic acid	91	$C_6H_6O_6$	$C_{15}H_{30}O_6Si_3$	55530-71-7	390.1345		375.1115	375.1132	12.20
P64	35.18	Amino acid	DL-Ornithine	93	$C_5H_{12}N_2O_2$	$C_{14}H_{36}N_2O_2Si_3$	1000333-14-1	348.2079	348.2104	333.1850		12.78

Code	Retention time (min)	Family	Compound name	Match factor	Formula	Derivatives of molecular formula	CAS	m/z (M^+)		m/z $(M\text{-}CH3)^+$		RSD (%, n=3)
								Theoretical	Experimental	Theoretical	Experimental	
P65	35.61	Ester	Phosphoric acid mono-(2,3-dihydroxy-propyl) ester	92	$C_3H_9O_6P$	$C_{15}H_{41}O_6PSi_4$	31038-11-6	460.1712		445.1483	445.1516	10.85
P66	35.73	Amine	L-Glutamine	94	$C_5H_{10}N_2O_3$	$C_{14}H_{34}N_2O_3Si_3$	70591-28-5	362.1872	362.1897	347.1642		10.62
P67	35.95	Organic acid	Terephthalic acid	88	$C_8H_6O_4$	$C_{14}H_{22}O_4Si_2$	4147-84-6	310.1051	310.1062	295.0822		9.60
P68	35.98	Organic acid	3,4,5,6-Tetrahydroxy-2-oxo-hexanoic acid	81	$C_6H_{15}O_7$	$C_{21}H_{50}O_7Si_5$	1000059-82-4	554.2397		539.2168		12.32
P69	36.02	Amine	Phosphorylethanolamine	94	$C_2H_8NO_4P$	$C_{14}H_{40}NO_4PSi_4$	55334-92-4	429.1767		414.1537		15.59
P70	36.38	Purine derivative	9H-Purin-6-ol	89	$C_5H_4N_4O$	$C_{11}H_{20}N_4OSi_2$	17962-89-9	280.1170	280.1183	265.0941		14.26
P71	36.83	Organic acid	Citric acid	88	$C_6H_8O_7$	$C_{18}H_{40}O_7Si_4$	14330-97-3	480.1846		465.1616	465.1647	12.02
P72	36.98	Organic acid	Myristic acid	83	$C_{14}H_{28}O_2$	$C_{17}H_{36}O_2Si$	18603-17-3	300.2479	300.2490	285.2250		9.90
P73	37.02	Amine	Cadaverine	82	$C_5H_{14}N_2$	$C_{17}H_{46}N_2Si_4$	65898-76-2	390.2733		375.2503	375.2520	14.61
P74	38.02	Amino acid	DL-tyrosine	90	$C_9H_{11}NO_3$	$C_{15}H_{27}NO_3Si_2$	7536-83-6	325.1524		310.1294	310.1311	11.92
P75	38.09	Sugar	D-Fructose	87	$C_6H_{15}NO_6$	$C_{22}H_{55}NO_6Si_5$	56196-14-6	569.2870		554.2641		7.35
P76	38.39	Sugar	d-Glucose	82	$C_6H_{12}O_6$	$C_{22}H_{55}NO_6Si_5$	130405-10-6	569.2870		554.2640		7.30
P77	38.47	Sugar	Galactopyranose	87	$C_6H_{12}O_6$	$C_{21}H_{52}O_6Si_5$	1769-00-2	540.2605		525.2375		12.30
P78	38.67	Sugar	D-Allose	93	$C_7H_{15}NO_6$	$C_{22}H_{55}NO_6Si_5$	1000380-19-7	569.2870		554.2640		6.01
P79	38.82	Amino acid	L-Lysine	81	$C_6H_{14}N_2O_2$	$C_{18}H_{46}N_2O_2Si_4$	55429-07-7	434.2631	434.2663	419.2401		9.53
P80	38.87	Amino acid	L-Histidine	81	$C_6H_9N_3O_2$	$C_{15}H_{33}N_3O_2Si_3$	17908-25-7	371.1875	371.1901	356.1646		10.29
P81	39.18	Amino acid	L-Tyrosine	92	$C_3H_{11}NO_3$	$C_{18}H_{35}NO_3Si_3$	51220-73-6	397.1919		382.1690	382.1714	9.75
P82	40.15	Sugar	Glucopyranose	94	$C_6H_{12}O_6$	$C_{21}H_{52}O_6Si_5$	1000157-49-7	540.2605		525.2375		15.98
P83	40.56	Organic acid	9-Hexadecenoic acid	93	$C_{16}H_{30}O_2$	$C_{19}H_{38}O_2Si$	1000333-19-8	326.2636		311.2406	311.2425	11.82
P84	40.78	Organic acid	D-Gluconic acid	90	$C_6H_{12}O_7$	$C_{24}H_{60}O_7Si_6$	34290-52-3	628.2949		613.2720		11.29
P85	40.94	Organic acid	Palmitic Acid	93	$C_{16}H_{32}O_2$	$C_{19}H_{40}O_2Si$	55520-89-3	328.2792	328.2815	313.2563		10.90
P86	41.23	Polyol	Scyllo-Inositol	88	$C_6H_{12}O_6$	$C_{24}H_{60}O_6Si_6$	14251-18-4	612.3000		597.2770		13.00

Code	Retention time (min)	Family	Compound name	Match factor	Formula	Derivatives of molecular formula	CAS	m/z (M^+)		m/z (M-CH3)$^+$		RSD (%, n=3)
								Theoretical	Experimental	Theoretical	Experimental	
P87	42.00	Organic acid	Ferulic acid	84	$C_{10}H_{10}O_4$	$C_{16}H_{26}O_4Si_2$	10517-09-6	338.1364	338.1380	323.1135		14.57
P88	42.35	Organic acid	Lanthionine	87	$C_6H_{12}N_2O_4S$	$C_{18}H_{44}N_2O_4SSi_4$	73200-54-1	496.2093		481.1864		9.36
P89	42.43	Polyol	Myo-Inositol	80	$C_6H_{12}O_6$	$C_{24}H_{60}O_6Si_6$	2582-79-8	612.3000		597.2770		8.17
P90	44.05	Organic acid	Linoleic acid	95	$C_{18}H_{32}O_2$	$C_{21}H_{40}O_2Si$	56259-07-5	352.2792	352.2816	337.2563		11.05
P91	44.06	Organic acid	Kynurenine	80	$C_{10}H_{12}N_2O_3$	$C_{19}H_{36}N_2O_3Si_3$	72088-03-0	424.2028	424.2058	409.1799		11.83
P92	44.15	Organic acid	Oleic acid	96	$C_{18}H_{34}O_2$	$C_{21}H_{42}O_2Si$	21556-26-3	354.2949	354.2975	339.2719		10.28
P93	44.37	Thioether	Cystathionine	87	$C_7H_{14}N_2O_4S$	$C_{19}H_{46}N_2O_4SSi_4$	1000141-33-0	510.2250	510.2263	495.2020		15.83
P94	44.53	Organic acid	Stearic acid	90	$C_{18}H_{36}O_2$	$C_{21}H_{44}O_2Si$	18748-91-9	356.3105	356.3129	341.2876		10.84
P95	44.59	Amino acid	L-Tryptophan	92	$C_{11}H_{12}N_2O_2$	$C_{20}H_{36}N_2O_2Si_3$	55429-28-2	420.2079		405.1850		12.94
P96	44.87	Polyol	D-Glucitol	86	$C_6H_{10}O_4$	$C_{24}H_{62}O_6Si_6$	14199-80-5	614.3157		599.2927		11.68
P97	51.72	Purine derivative	Adenosine	91	$C_{10}H_{13}N_5O_4$	$C_{22}H_{45}N_5O_4Si_4$	53294-33-0	555.2543		540.2314	540.2352	10.19
P98	52.36	Sugar	Sucrose	96	$C_{12}H_{22}O_{11}$	$C_{36}H_{86}O_{11}Si_8$	19159-25-2	918.4319		903.4089		9.02

Table S3. The concentrations of identified metabolites in different products.

Compound No.	Retention time (min)	Compound name	Concentration of identified metabolites (mg/kg)																
			WCF	CCF	LCF0	LCF1	LCF2	LCF3	LCF4	LFB	LOS1	LOS2	LOS3	LOS4	LOS5	TxLU	TxLI	TxPU	TxPI
P1	14.16	Acetylacetone	22.11	37.30	31.47	25.92	38.20	50.20	47.50	45.39	28.76	4.60	9.45	20.78	13.22	3.41	4.33	7.01	0.65
P2	14.29	N,N-Dimethylglycine	243.17	208.43	153.30	121.60	228.74	269.58	215.45	701.50	36.73	21.59	21.72	112.13	10.74	1.90	3.02	7.96	5.10
P3	14.37	Methoxyimino-acetic acid	17.73	17.44	21.30	15.31	20.36	20.77	36.86	30.05	111.42	85.59	96.46	126.09	132.45	39.53	30.91	185.49	103.89
P4	14.46	Boric acid	118.32	184.00	169.60	107.53	180.39	148.54	491.75	139.57	317.78	128.68	237.14	192.67	220.94	471.49	432.11	212.15	226.31
P5	14.48	2,3,6-Trimethyl-pyridine	159.11	157.34	132.39	104.01	150.90	153.69	132.38	174.04	3.28	1.92	3.17	0.67	2.56	1.14	0.41	126.94	112.44
P6	15.89	tert-Butyl carbamate	197.31	337.76	202.47	131.37	298.57	301.32	408.68	404.55	54.55	54.96	64.35	43.42	75.91	270.21	293.26	173.80	105.89
P7	16.38	2-Methoxyimino-propionic acid	18.15	14.64	9.43	8.29	13.77	17.93	19.10	31.23	85.67	63.59	19.80	16.86	7.40	4234.35	5204.40	388.32	149.14
P8	16.53	Diethyl-carbamic acid	293.97	281.61	241.37	180.79	273.17	262.05	233.23	301.11	973.07	928.75	984.63	791.82	1038.96	1485.06	1425.82	249.14	208.57
P9	16.74	Lactic Acid	262.84	134.52	26.92	19.01	33.21	31.40	52.64	39.80	287.25	393.74	324.14	481.33	294.24	1018.86	1014.22	6045.37	6573.05
P10	17.08	Butanedioic acid, dimethyl ester	440.52	737.82	349.28	162.96	631.66	581.58	571.28	441.59	178.24	188.23	161.55	739.67	243.65	170.86	351.95	312.12	95.12
P11	17.18	Glycolic acid	29.17	13.43	6.77	6.31	8.94	9.29	8.06	25.90	27.95	31.68	33.72	32.53	31.23	8.93	10.49	60.75	15.04
P12	17.86	L-Valine	4.37	0.83	5.61	6.57	8.40	12.50	8.47	0.40	97.13	92.80	83.76	85.32	76.67	2.05	2.98	9.83	9.12
P13	18.06	L-Alanine	1532.95	683.85	272.41	126.94	358.38	396.47	256.80	298.76	272.02	302.56	363.16	245.45	316.11	3453.44	3216.76	3463.97	2725.67
P14	19.01	Glycine	481.51	341.67	560.37	549.46	198.61	408.40	366.07	4001.34	9299.10	12954.94	21889.46	9604.60	38851.59	268.04	133.05	6760.30	3057.74
P15	19.06	3-Pyridinol	8765.55	11994.33	11396.86	9214.47	12249.28	12955.70	13077.65	8853.46	5984.45	5903.30	6098.06	6715.71	6141.78	6682.80	6752.34	8544.07	7206.75
P16	19.23	3-Mercapto-propionic acid	252.56	185.34	138.16	97.80	165.00	148.30	157.32	256.05	46.63	55.58	52.09	36.25	59.11	229.32	234.29	176.03	158.22
P17	19.53	4-Pyridinol	228.87	332.42	288.62	217.05	350.83	385.92	425.83	964.46	65.32	86.56	56.59	43.20	63.01	150.60	143.90	216.46	160.07
P18	19.80	3-Hydroxy-2-methyl-propionic acid	7.17	5.63	3.23	1.81	3.15	4.02	4.77	6.41	5.44	6.97	6.70	7.41	6.24	6.12	5.29	112.41	55.62
P19	20.19	2-Aminobutanoic acid	2114.87	1304.48	617.31	393.20	1090.17	1576.69	1229.56	2328.01	46.71	113.48	40.49	142.16	179.82	581.68	996.38	1718.48	1102.83
P20	20.39	Phosphoric acid monomethyl ester	179.90	1031.86	967.18	755.76	1322.20	1382.37	2067.30	405.24	102.95	57.35	46.45	45.19	45.20	276.93	149.12	670.34	254.98
P21	21.27	3-Aminoisobutyric acid	123.05	201.42	155.71	86.81	198.09	196.39	174.26	12.56	13.94	13.71	13.57	12.77	14.02	90.74	116.91	316.33	181.92
P22	21.98	4-Hydroxybutanoic acid	35.59	17.43	1.01	0.61	1.63	0.97	1.01	2.02	7.13	7.07	0.35	0.88	5.82	97.41	102.40	218.66	176.86

Compound No.	Retention time (min)	Compound name	Concentration of identified metabolites (mg/kg)																
			WCF	CCF	LCF0	LCF1	LCF2	LCF3	LCF4	LFB	LOS1	LOS2	LOS3	LOS4	LOS5	TxLU	TxLI	TxPU	TxPI
P23	22.19	2-Amino-3-methyl-butyric acid	4556.04	6253.25	14957.70	12834.32	4776.84	5902.09	4727.77	14237.23	992.65	1306.63	909.45	956.69	822.80	5074.36	5417.07	12341.94	14932.24
P24	22.68	L-Serine	461.03	380.59	367.16	362.33	418.20	495.85	297.70	1156.61	62239.54	66830.66	67186.83	61732.64	62623.95	1416.82	838.87	1642.15	1137.64
P25	22.99	Ethanolamine	5486.70	14613.05	4884.95	2717.61	8168.20	6261.64	5748.89	5169.70	2031.49	1825.74	2003.12	1389.81	1216.37	2813.30	4329.33	13157.66	15314.62
P26	23.14	L-Leucine	6339.12	7025.40	14.37	13.62	6990.66	5541.88	5241.97	13.97	3702.95	1188.12	4180.09	3018.31	3833.31	10316.26	4623.58	5746.90	13696.77
P27	23.28	Glycerol	11092.81	3271.00	1044.27	1142.15	1850.21	1112.01	1726.57	189.35	10068.92	12084.80	2713.29	10666.96	9719.46	14297.52	15522.63	14790.47	18679.14
P28	23.67	Niacin	451.76	71.76	66.07	57.75	95.01	69.81	58.72	131.82	2403.55	2724.46	2438.08	2436.68	2353.37	140.22	176.08	856.14	517.96
P29	23.88	L-Proline	1510.97	1309.66	965.06	736.53	1324.62	1599.71	1478.36	2014.04	89.89	53.09	45.47	47.77	68.02	7217.87	7852.28	3061.73	1687.94
P30	24.84	Glyceric acid	293.12	131.03	46.02	31.62	75.55	54.88	47.09	130.79	445.49	474.92	502.38	517.95	484.43	14.33	21.40	53.08	37.62
P31	25.01	Uracil	188.87	98.82	61.38	44.13	142.93	72.92	85.03	126.71	231.49	208.21	235.58	213.22	197.31	161.05	133.52	5866.79	4126.95
P32	25.07	E-2-Butenedioic acid	910.15	1196.15	540.58	390.40	945.92	1400.62	1280.29	1909.46	13798.90	14488.33	10939.72	7229.46	4797.94	26.04	25.55	175.14	245.85
P33	25.67	DL-Serine	6311.68	7493.97	1564.24	6254.15	6435.23	6909.08	5763.74	7963.18	4.12	4.66	3.71	7.24	7.93	7878.86	8685.83	12876.62	8628.52
P34	26.39	Isopropyl formate	9529.63	11763.25	10448.76	6342.61	11233.07	12631.63	12001.17	5587.11	0.00	0.00	0.00	0.00	0.00	10826.84	11731.32	10767.83	8422.46
P35	26.39	L-Threonine	2924.09	3831.47	3272.19	1829.34	3511.11	4430.95	3868.68	1438.79	99.46	109.30	2.88	86.14	3.83	3567.80	3994.00	3545.48	2565.63
P36	26.62	Pentanedioic acid	14.39	5.94	4.55	3.49	5.06	6.14	8.35	3.81	11.74	0.84	1.56	0.57	1.05	2.89	4.73	331.15	48.77
P37	26.87	L-Methionine	0.46	1.04	0.75	1.67	0.49	0.42	0.17	1.70	218.73	469.59	162.08	358.00	273.90	0.46	0.26	27.15	24.48
P38	27.36	β-Alanine	523.42	595.61	266.67	124.29	329.50	363.99	377.28	355.08	143003.83	204567.98	108996.13	177710.15	783.25	7691.04	12693.48	7897.27	4436.54
P39	27.85	2,2'-Bipyridine	50.57	94.82	76.45	61.73	97.74	116.71	101.31	267.79	284.88	37.48	21.74	37.07	47.19	15.77	6.33	49.73	34.51
P40	27.98	Homoserine	559.46	261.97	101.15	100.02	311.18	335.65	331.37	1949.09	2.64	4.56	3.97	2.78	7.33	9.32	171.83	72.48	53.13
P41	28.16	3-Amino-2-piperidone	841.32	3196.20	2834.81	1125.34	3448.69	4111.60	3041.25	116.89	0.41	3.45	6.95	8.44	9.56	758.25	504.90	2529.71	1061.87
P42	28.55	Aminomalonic acid	103.33	46.59	36.44	15.85	42.79	29.22	16.70	163.14	5331.36	4529.33	4620.47	4026.48	4148.23	521.54	405.20	210.22	104.08
P43	28.99	DL-malic acid	1250.27	2003.09	1091.34	829.23	1870.60	2436.24	2719.45	3103.46	30824.21	30833.39	31150.41	31157.06	31692.57	361.80	467.90	2077.42	1482.09
P44	29.60	Erythritol	151.01	27.73	15.22	26.00	60.84	26.21	26.99	134.86	24.43	60.14	51.03	56.97	97.01	32.44	43.41	244.13	136.34
P45	29.78	L-Aspartic acid	11968.22	20104.03	16043.77	12478.89	18409.32	20146.47	18597.29	22059.37	1389.51	2286.80	472.69	944.59	744.36	45.72	6.75	1623.02	126.09
P46	29.88	L-5-Oxoproline	16305.61	19368.22	15389.81	9584.53	19017.66	18122.82	16605.36	19517.20	1.77	1.94	2.14	1.51	1.83	5744.69	7243.63	10035.62	2874.21

Compound No.	Retention time (min)	Compound name	Concentration of identified metabolites (mg/kg)																
			WCF	CCF	LCF0	LCF1	LCF2	LCF3	LCF4	LFB	LOS1	LOS2	LOS3	LOS4	LOS5	TxLU	TxLI	TxPU	TxPI
P47	29.99	4-Aminobutanoic acid	5785.50	1416.50	1830.01	2129.51	4167.39	1403.13	1493.44	787.30	41851.10	40032.56	41598.44	42607.26	42055.97	80.82	103.13	1190.29	1599.43
P48	30.39	DL-Phenylalanine	281.38	340.01	242.86	265.89	446.98	470.38	411.77	937.44	47.90	28.76	33.35	402.95	69.09	60.75	38.91	303.97	297.56
P49	30.66	Cysteine	131.37	177.32	123.03	72.67	212.39	183.05	182.47	126.45	0.47	0.77	1.28	0.96	2.14	849.21	861.10	941.99	562.50
P50	30.91	2,3,4-Trihydroxy-butanoic acid	671.50	163.44	128.99	98.20	179.70	159.08	128.88	419.70	956.48	950.29	1061.77	978.13	884.56	140.32	134.18	346.59	216.98
P51	31.07	2-Hydroxyglutaric acid	406.75	80.32	15.60	12.10	16.26	13.19	12.09	17.66	1.39	3.74	4.15	9.04	4.70	1383.53	1583.40	6019.29	3653.59
P52	31.57	L-Asparagine	242.43	625.91	451.65	315.14	899.51	912.14	921.28	1818.95	0.64	0.76	0.98	0.69	0.54	130.06	165.34	149.36	74.43
P53	31.77	Pipecolic acid	1553.82	3004.02	80.23	1132.03	2107.97	143.26	153.46	243.89	8.28	2.23	2.11	3.33	0.73	57.06	77.59	520.36	232.86
P54	32.03	L-Ornithine	516.89	1797.56	1698.17	730.58	1892.34	1953.81	1865.38	45.26	0.05	0.15	0.12	0.12	0.00	493.23	334.97	1392.58	723.82
P55	32.15	L-Glutamic acid	28857.78	35500.53	27846.04	23064.23	32456.34	35041.60	27845.51	34562.72	1.32	6.78	1.33	0.85	1.15	5316.13	5620.55	10949.31	7911.16
P56	32.38	L-Phenylalanine	9839.63	13737.99	11155.84	9338.63	13968.49	14297.18	14247.12	10611.38	31.43	35.45	38.21	39.91	34.97	5663.68	4511.82	14136.48	13608.45
P57	33.16	3,4,5-Trihydroxy-pentanoic acid	2.34	5.48	4.14	3.80	4.57	5.97	3.35	314.61	0.37	0.33	3.51	0.28	0.67	2.25	2.61	19.20	12.80
P58	33.41	Asparagine	2334.36	5575.97	4583.94	2799.16	5828.03	6408.06	6124.30	2778.28	0.93	0.31	0.51	0.64	0.56	2690.96	4221.59	2001.40	1330.91
P59	33.76	2,3,4,5-Tetrahydroxy-pentanal O-ethyl-oxime	15.96	30.10	32.25	21.01	34.73	29.31	18.03	56.63	100.75	4.45	45.75	75.48	101.22	177.58	121.10	81.36	36.91
P60	34.34	2-Aminoadipic acid	289.22	704.83	763.34	297.50	687.63	760.86	784.22	1155.59	0.13	0.21	0.64	0.08	0.57	14.54	23.51	1478.57	256.39
P61	34.73	Xylitol	10712.16	20867.99	16126.88	12776.73	18268.51	19984.38	20284.90	18442.97	7598.60	8539.28	8390.36	8705.52	8197.56	12505.46	12885.73	13072.37	11587.16
P62	34.91	Putrescine	12195.05	12273.86	1655.00	3024.44	10909.86	12603.41	15668.91	14691.14	8606.56	9194.46	10586.34	12166.86	7372.32	2583.04	1052.56	6223.38	14049.22
P63	35.01	Aconitic acid	25.46	20.64	22.26	22.07	24.69	30.76	42.58	32.19	1.84	0.57	5.09	0.84	0.17	3.28	4.16	2.99	2.30
P64	35.18	DL-Ornithine	2098.24	3335.98	1339.20	1038.40	2990.24	3202.08	3451.15	1320.83	0.44	0.42	0.78	0.44	0.22	94.71	43.61	1790.31	723.30
P65	35.61	Phosphoric acid mono-(2,3-dihydroxy-propyl) ester	294.36	279.96	491.86	274.70	435.14	514.09	410.76	2305.28	2063.41	219.39	659.19	285.15	328.76	4810.68	5094.94	2279.57	1575.77
P66	35.73	L-Glutamine	5631.40	16321.25	16371.82	11986.29	18541.64	20294.21	20344.21	8071.05	0.34	1.07	0.39	0.19	0.20	3792.40	6215.85	5291.47	3045.89
P67	35.95	Terephthalic acid	502.71	574.63	537.89	582.45	587.20	622.71	880.55	640.07	622.66	147.23	218.73	79.81	80.11	544.01	772.35	452.35	444.12
P68	35.98	3,4,5,6-Tetrahydroxy-2-oxo-hexanoic acid	204.72	351.86	249.04	219.49	379.05	405.78	666.64	866.99	9727.10	2840.68	4668.45	4138.34	4855.12	48.83	87.90	236.99	137.55
P69	36.01	Phosphorylethanolamine	102.37	1634.70	2931.86	1605.33	139.91	182.59	133.94	12485.88	7.49	1.29	4.48	7.15	2.51	9874.80	12767.53	11303.51	4286.24

Compound No.	Retention time (min)	Compound name	Concentration of identified metabolites (mg/kg)																
			WCF	CCF	LCF0	LCF1	LCF2	LCF3	LCF4	LFB	LOS1	LOS2	LOS3	LOS4	LOS5	TxLU	TxLI	TxPU	TxPI
P70	36.38	9H-Purin-6-ol	114.96	4.31	6.56	1.67	7.28	9.25	5.22	2.09	0.34	0.32	0.30	0.32	0.43	279.63	195.16	2820.50	2102.10
P71	36.83	Citric acid	21190.31	20924.73	17769.12	15133.83	20511.18	20386.00	18245.51	24732.85	1.78	0.97	0.78	1.88	0.82	0.38	0.22	0.46	0.26
P72	36.98	Myristic acid	42.13	46.50	35.59	43.93	56.65	25.57	43.87	15.16	52.40	39.91	46.67	40.67	42.91	138.78	164.38	295.92	670.27
P73	37.02	Cadaverine	2445.17	2824.17	2283.95	1334.26	3592.68	3839.01	3465.31	1530.48	7.22	16.63	6.71	8.06	25.76	1362.94	1386.34	4282.13	10462.92
P74	38.02	DL-tyrosine	355.41	454.28	375.70	183.77	613.74	560.94	475.43	1094.02	1.27	6.99	1.93	1.40	4.72	959.28	795.78	5123.78	3433.78
P75	38.08	D-Fructose	1723.37	266.35	85.97	54.33	127.70	76.75	126.54	89.27	45026.35	53471.19	47418.77	44990.92	41529.82	50.51	444.16	281.06	135.10
P76	38.67	d-Glucose	1390.82	832.82	322.79	284.83	14702.66	391.92	454.86	369.70	120541.80	133368.73	129054.90	131409.37	128657.98	7715.15	8829.20	739.80	23487.71
P77	38.47	Galactopyranose	229.96	103.24	140.54	83.75	293.78	386.22	405.00	247.41	1153.49	1386.45	1254.54	1187.91	1098.38	640.18	924.12	7195.75	6555.78
P78	38.67	D-Allose	19720.57	19175.25	24047.46	26641.55	17420.42	27377.77	29133.90	22416.94	36535.32	80168.87	74939.69	73045.63	64340.92	30980.40	46521.74	41275.54	39919.92
P79	38.82	L-Lysine	10029.60	10250.61	8501.14	6183.49	10254.60	11888.73	10124.79	1056.66	0.43	0.96	0.39	0.91	1.25	6669.24	7739.61	12834.86	9868.40
P80	38.87	L-Histidine	4348.25	5162.31	2939.64	1393.82	4970.13	6146.82	4784.36	482.24	0.33	0.16	0.26	0.10	0.12	12161.89	12944.06	5995.92	3642.69
P81	39.17	L-Tyrosine	7966.71	8067.29	5219.77	20836.79	7820.11	8505.39	8371.09	8577.95	26.61	59.09	60.80	85.90	97.05	12831.33	12280.36	13581.85	16467.25
P82	40.15	Glucopyranose	103.63	270.28	192.47	28.26	428.45	452.90	334.43	356.48	12836.21	12986.77	13471.13	9091.49	11773.55	1385.67	874.13	11013.70	10885.12
P83	40.56	9-Hexadecenoic acid	194.42	140.42	59.97	109.55	173.66	85.30	51.90	32.39	2.87	7.09	9.27	15.48	6.82	189.51	203.81	484.70	913.89
P84	40.78	D-Gluconic acid	746.91	150.25	30.37	21.51	63.65	36.09	51.07	295.11	718.52	146.97	225.23	68.43	90.79	13.11	24.90	423.84	155.87
P85	41.23	Palmitic Acid	291.93	149.40	218.87	92.45	171.47	180.11	147.61	686.54	126.65	202.30	241.95	246.24	149.58	27.32	312.92	12.10	31.30
P86	40.93	Scyllo-Inositol	13311.44	9891.43	7834.41	12556.99	14316.67	3734.76	4281.54	2657.99	644.13	416.68	640.23	411.60	354.01	12213.29	13576.19	19338.88	28627.49
P87	42.00	Ferulic acid	2.22	4.65	20.74	5.06	5.11	7.34	4.20	0.86	0.15	0.22	0.13	0.12	0.16	24.88	47.03	44.04	36.79
P88	42.35	Lanthionine	99.57	248.40	185.73	41.05	143.93	261.14	214.40	115.53	2.77	3.14	7.31	7.01	7.71	2895.63	3204.97	8211.66	8360.68
P89	42.43	Myo-Inositol	2124.50	398.19	170.97	116.00	253.71	226.77	248.60	2774.01	34651.13	34944.30	34210.67	37001.41	38050.68	503.56	571.80	3559.01	2612.09
P90	44.05	Linoleic acid	4855.54	2054.74	920.87	1191.56	2088.68	1460.48	975.29	789.20	43.16	7.08	15.01	30.54	23.04	1373.38	1703.47	5809.00	6684.59
P91	44.06	Kynurenine	0.54	0.34	0.16	0.09	0.62	0.21	0.69	0.28	1.25	1.33	0.67	1.07	1.13	0.14	0.15	1358.64	610.28
P92	44.15	Oleic acid	9270.78	7171.84	4950.50	5475.21	6998.13	4977.68	4706.69	98.53	1.52	8.68	17.23	18.19	1.36	6892.74	8622.29	19360.47	27063.37
P93	44.37	Cystathionine	187.77	385.54	234.83	104.44	228.85	414.51	356.29	131.81	0.28	0.28	0.21	0.35	0.27	6.16	15.67	198.92	208.97

Compound No.	Retention time (min)	Compound name	Concentration of identified metabolites (mg/kg)																
			WCF	CCF	LCF0	LCF1	LCF2	LCF3	LCF4	LFB	LOS1	LOS2	LOS3	LOS4	LOS5	TxLU	TxLI	TxPU	TxPI
P94	44.53	Stearic acid	1101.53	520.76	400.76	403.16	597.28	287.31	2383.00	380.49	58.26	52.29	64.27	50.32	42.51	1035.17	1107.71	2333.56	2765.54
P95	44.59	L-Tryptophan	1179.09	874.31	1129.81	576.92	780.39	925.50	932.29	798.81	0.82	0.51	0.13	0.21	0.41	4051.28	1079.99	9889.36	11973.24
P96	44.87	D-Glucitol	1042.83	343.65	180.28	160.20	439.05	300.01	256.53	457.92	3.52	4.36	2.57	1.31	3.72	0.88	14.78	1.45	1.44
P97	51.72	Adenosine	1523.79	407.61	72.90	39.67	141.56	70.67	51.01	137.81	11659.50	12798.46	11581.88	11586.94	10373.55	87.71	99.75	35.24	73.53
P98	52.36	Sucrose	71441.04	59447.05	18659.10	31148.67	50631.48	52056.58	50751.48	70687.33	29610.89	231855.81	198712.56	247340.01	209603.44	54021.23	52875.95	8817.60	8846.48

Table S4. Top ten compounds in concentrations from different products.

Sample	1		2		3		4		5		6		7		8		9		10	
	Compound No.	Content (mg/kg)	Compound No.	Content (mg/kg)	Compound No.	Content (mg/kg)	Compound No.	Content (mg/kg)	Compound No.	Content (mg/kg)	Compound No.	Content (mg/kg)	Compound No.	Content (mg/kg)	Compound No.	Content (mg/kg)	Compound No.	Content (mg/kg)	Compound No.	Content (mg/kg)
WCF	P98	71441.04	P55	28857.78	P71	21190.31	P78	19720.57	P46	16305.61	P86	13311.44	P62	12195.05	P45	11968.22	P27	11092.81	P61	10712.16
CCF	P98	59447.05	P55	35500.53	P71	20924.73	P61	20867.99	P45	20104.03	P46	19368.22	P78	19175.25	P66	16321.25	P25	14613.05	P56	13737.99
LCF0	P55	27846.04	P78	24047.46	P98	18659.10	P71	17769.12	P66	16371.82	P61	16126.88	P45	16043.77	P46	15389.81	P23	14957.70	P15	11396.86
LCF1	P98	31148.67	P78	26641.55	P55	23064.23	P81	20836.79	P71	15133.83	P23	12834.32	P61	12776.73	P86	12556.99	P45	12478.89	P66	11986.29
LCF2	P98	50631.48	P55	32456.34	P71	20511.18	P46	19017.66	P66	18541.64	P45	18409.32	P61	18268.51	P78	17420.42	P76	14702.66	P86	14316.67
LCF3	P98	52056.58	P55	35041.60	P78	27377.77	P71	20386.00	P66	20294.21	P45	20146.47	P61	19984.38	P46	18122.82	P56	14297.18	P15	12955.70
LCF4	P98	50751.48	P78	29133.90	P55	27845.51	P66	20344.21	P61	20284.90	P45	18597.29	P71	18245.51	P46	16605.36	P62	15668.91	P56	14247.12
LFB	P98	70687.33	P55	34562.72	P71	24732.85	P78	22416.94	P45	22059.37	P46	19517.20	P61	18442.97	P62	14691.14	P23	14237.23	P69	12485.88
LOS1	P38	143003.83	P76	120541.80	P24	62239.54	P75	45026.35	P47	41851.10	P78	36535.32	P89	34651.13	P43	30824.21	P98	29610.89	P32	13798.90
LOS2	P98	231855.81	P38	204567.98	P76	133368.73	P78	80168.87	P24	66830.66	P75	53471.19	P47	40032.56	P89	34944.30	P43	30833.39	P32	14488.33
LOS3	P98	198712.56	P76	129054.90	P38	108996.13	P78	74939.69	P24	67186.83	P75	47418.77	P47	41598.44	P89	34210.67	P43	31150.41	P14	21889.46
LOS4	P98	247340.01	P38	177710.15	P76	131409.37	P78	73045.63	P24	61732.64	P75	44990.92	P47	42607.26	P89	37001.41	P43	31157.06	P62	12166.86
LOS5	P98	209603.44	P76	128657.98	P78	64340.92	P24	62623.95	P47	42055.97	P75	41529.82	P14	38851.59	P89	38050.68	P43	31692.57	P82	11773.55
TxLU	P98	54021.23	P78	30980.40	P27	14297.52	P81	12831.33	P61	12505.46	P86	12213.29	P80	12161.89	P34	10826.84	P26	10316.26	P69	9874.80
TxLI	P98	52875.95	P78	46521.74	P27	15522.63	P86	13576.19	P80	12944.06	P61	12885.73	P69	12767.53	P38	12693.48	P81	12280.36	P34	11731.32
TxPU	P78	41275.54	P92	19360.47	P86	19338.88	P27	14790.47	P56	14136.48	P81	13581.85	P25	13157.66	P61	13072.37	P33	12876.62	P79	12834.86
TxPI	P78	39919.92	P86	28627.49	P92	27063.37	P76	23487.71	P27	18679.14	P81	16467.25	P25	15314.62	P23	14932.24	P62	14049.22	P26	13696.77

Table S5. Results of Significance Analysis of Metabolomics (SAM).

Metabolite	d.value	stdev	rawp	q.value
Citric acid	38.866	0.016437	0	0
L-Glutamine	30.606	0.051326	0	0
L-Histidine	28.556	0.10068	0	0
L-Glutamic acid	26.218	0.042574	0	0
L-Lysine	24.693	0.078012	0	0
L-5-Oxoproline	23.377	0.017388	0	0
Asparagine	22.788	0.034207	0	0
L-Ornithine	22.248	0.24214	0	0
L-Tryptophan	20.787	0.08216	0	0
DL-Ornithine	17.31	0.15427	0	0
DL-Serine	15.18	0.034172	0	0
2-Aminoadipic acid	13.695	0.25498	0	0
L-Asparagine	12.109	0.048584	0	0
Oleic acid	11.648	0.31021	0	0
Cystathionine	10.798	0.15555	0	0
Cysteine	9.8191	0.030501	0	0
L-Phenylalanine	9.7942	0.017557	0	0
Phosphorylethanolamine	9.6259	0.34371	0	0
DL-tyrosine	9.4954	0.090277	0	0
9H-Purin-6-ol	9.48	0.23463	0	0
D-Fructose	8.735	0.15007	0	0
3-Amino-2-piperidone	8.697	0.24668	0	0
Lanthionine	8.6905	0.062647	0	0
Cadaverine	8.6409	0.072732	0	0
D-Glucitol	7.9029	0.11047	0	0
L-Tyrosine	7.8836	0.028593	0	0
L-Methionine	7.6682	0.31912	0	0
2-Hydroxyglutaric acid	7.2319	0.19349	0	0
L-Serine	6.6673	0.019619	0	0
Pipecolic acid	6.2123	0.27263	0	0
Linoleic acid	6.0012	0.086702	0.000102	4.33E-05
L-Threonine	5.9667	0.20214	0.000102	4.33E-05
Adenosine	5.958	0.14836	0.000102	4.33E-05
d-Glucose	5.7383	0.2496	0.000306	0.000116
Ferulic acid	5.6921	0.085019	0.000306	0.000116
L-Aspartic acid	5.5494	0.23511	0.000306	0.000116
b-Alanine	5.5343	0.33275	0.000306	0.000116
Aminomalonic acid	5.4269	0.083905	0.000408	0.00015
Myo-Inositol	4.7294	0.17018	0.001122	0.000403

Table S6. Predictions of pathways involving featured metabolites from Figure 4C.

Pathway Name	Match Status	p	-log(p)	Holm p	FDR	Impact	Details
Alanine, aspartate and glutamate metabolism	3/23	0.0049299	2.3072	0.39932	0.21126	0.42906	KEGG
Glyoxylate and dicarboxylate metabolism	3/24	0.0055783	2.2535	0.44627	0.21126	0.0875	KEGG
Galactose metabolism	3/27	0.0078243	2.1066	0.61812	0.21126	0.0	KEGG
Arginine biosynthesis	2/12	0.014443	1.8404	1.0	0.29246	0.05714	KEGG
beta-Alanine metabolism	2/14	0.019542	1.709	1.0	0.31658	0.277	KEGG
Aminoacyl-tRNA biosynthesis	3/48	0.037536	1.4255	1.0	0.44627	0.0	KEGG
Citrate cycle (TCA cycle)	2/20	0.038567	1.4138	1.0	0.44627	0.14041	KEGG
Glutathione metabolism	2/26	0.062368	1.205	1.0	0.62788	0.04167	KEGG
Nitrogen metabolism	1/5	0.077516	1.1106	1.0	0.62788	0.0	KEGG
D-Glutamine and D-glutamate metabolism	1/5	0.077516	1.1106	1.0	0.62788	1.0	KEGG
Glycerolipid metabolism	1/13	0.18989	0.7215	1.0	1.0	0.1875	KEGG
Butanoate metabolism	1/14	0.20299	0.69252	1.0	1.0	0.0	KEGG
Fructose and mannose metabolism	1/16	0.2286	0.64092	1.0	1.0	0.0459	KEGG
Pantothenate and CoA biosynthesis	1/18	0.25344	0.59613	1.0	1.0	0.0	KEGG
Propanoate metabolism	1/21	0.28928	0.53868	1.0	1.0	0.0	KEGG
Pyruvate metabolism	1/22	0.30087	0.52162	1.0	1.0	0.0	KEGG

Pathway Name	Match Status	p	-log(p)	Holm p	FDR	Impact	Details
Porphyrin and chlorophyll metabolism	1/24	0.32351	0.49012	1.0	1.0	0.0	KEGG
Arginine and proline metabolism	1/31	0.39742	0.40075	1.0	1.0	0.11228	KEGG
Pyrimidine metabolism	1/40	0.48132	0.31757	1.0	1.0	0.0	KEGG
Fatty acid biosynthesis	1/43	0.50675	0.29521	1.0	1.0	0.0	KEGG
